# Diagnostic value and disease evaluation significance of abdominal ultrasound inspection for neonatal necrotizing enterocolitis

**DOI:** 10.12669/pjms.325.10413

**Published:** 2016

**Authors:** Li Wang, Yinghui Li, Jian Liu

**Affiliations:** 1 Li Wang, Department of Special Inspection, Binzhou People’s Hospital, Shandong 256610, China; 2 Yinghui Li, Department of Health Examination, Binzhou People’s Hospital, Shandong 256610, China; 3 Jian Liu, Department of Special Inspection, Binzhou People’s Hospital, Shandong 256610, China

**Keywords:** Newborn, Neonatal Necrotizing Enterocolitis, Abdominal Ultrasound

## Abstract

**Objective:**

To summarize abdominal plain X-rays and ultrasound characteristics of 144 cases of Neonatal Necrotizing Enterocolitis (NEC) and to analyze diagnostic value and disease evaluation significance of abdominal ultrasound inspection for NEC.

**Methods:**

Clinical data of 144 NEC patients were retrospectively analyzed from February 2014 to December 2015. The patients were divided into suspected NEC group (N=74) and confirmed NEC group (N=70) according to amended Bell-NEC classification and diagnostic criteria. Meanwhile, we divided them into internal medicine treatment group (N=95) and surgery/death group (N=49) according to clinical prognosis and took records of their clinical manifestations, laboratory inspection results and abdominal plain X-rays and ultrasound characteristics.

**Results:**

For confirmed NEC group, the detection rate of portal venous gas (PVG) and dilatation of intestine by abdominal ultrasound was obviously higher than by plain X-rays (P<0.05). Abdominal ultrasound inspection revealed that the incidence rate of dilatation of intestine, bowel wall thickening and ascites (acoustic transmission difference) of the surgery/death group was higher than that of the internal medicine treatment group by comparing risk ratio (RR) and 95% confidence interval (CI) of RR; the difference was statistically significant (P<0.05). The abdominal plain X-rays inspection only showed the result that dilatation of intestine and free intraperitoneal air was more often found in the surgery/death group (P<0.05).

**Conclusion:**

Compared with abdominal plain X-rays, abdominal ultrasound has certain clinical value and offers more advantages in some aspects; therefore, it can be considered as the reference index in prediction of clinical prognosis.

## INTRODUCTION

Neonatal necrotizing enterocolitis (NEC) is a kind of acute intestinal necrotizing disease induced by unknown pathogeny and is also the most common disease which needs intervention of surgery in neonatal intensive care unit (ICU).^1^ Primary symptoms of NEC include abdominal distention, emesis and bloody stool. It is typically seen in premature infants with partial or extensive necrosis in small intestine and colon as its primary pathological manifestation.

Mild cases of NEC have favorable prognosis. The case fatality rate of low birth weight infant (LBWI) is up to 15%~30%.^2^,^3^ Part of infantile patients have unfavorable prognosis. A research indicated that the clinical prognosis of NEC was closely related to its severity.^4^ Thus, early diagnosis and timely treatment is the key factors which affect the clinical prognosis of NEC.

In recent decades, the research of diagnosis and treatment for NEC goes deeper while people get more and more acquaintance of it. The current diagnosis of NEC mainly relies on abdominal plain X-rays and observation of patients’ clinical manifestations.^5^ Abdominal plain X-rays is currently the standard imaging method for evaluating NEC; however, it has limitations. On the one hand it can neither observe dynamically nor detect intestinal necrosis and enterobrosis in time for it is discontinuously dynamic and it also cannot find existing enterobrosis which leads to disease exacerbation and delayed treatment; on the other hand repeated radiography will do more damage to infantile patients by causing more ionizing radiation.^6^,^7^ In recent year’s studies on abdominal ultrasound is gradually applied to early diagnosis of NEC and shows the advantages of convenience, real time and dynamic.^8^,^9^

Diagnostic value for NEC was earliest recognized in year 1984. Until year 2005 the importance of abdominal ultrasound for early diagnosis of NEC was gradually recognized by more and more researchers after the publication of feasibility study report (FSR) on evaluating infantile patients’ intestines by adopting color Doppler ultrasound (CDUS) and the summary report by Kim et al. in which it was found that the assessment of grey scale ultrasound of bowel wall was helpful to the diagnosis of NEC.^10^

To analyze the value of abdominal ultrasound in diagnosis and severity evaluation of NEC, this study collected the clinical data of 114 infants who were diagnosed as NEC, aiming to provide a theoretical basis for the diagnosis and treatment of NEC.

## METHODS

One hundred and forty-four NEC patients who were hospitalized in the hospital from Feb. 2014 to Dec. 2015 were selected for this study. Among them, 110 patients were males and 34 patients were females; the gestational age of those patients was from 25.1 to 40 weeks (average (33±3) weeks); there were 108 premature infants (75%) and 36 full-term infants (25%). Premature infants’ birth weight was from 740 to 4410 g (average (2045±798) g). Patients who were diagnosed as NEC according to the Practical Neonatology (the fourth edition) and the amended Bell-NEC classification and diagnostic criteria,^11^ developed NEC within thirty days after birth, have underwent abdominal plain X-ray examination and ultrasound (interval of two examinations less than 24 hour), had completed hospitalization data and whose parents had signed informed consent were included in the study. Patients who suffered from intestinal malformation (congenital megacolon, intestinal atresia, malrotation of intestines, etc.) or complicated congenital heart disease were excluded.

### Grouping

According to clinical manifestations of 144 NEC infantile patients, they were divided into suspected NEC group (N=74) and confirmed NEC group (N=70) with reference to the amended Bell-NEC classification and diagnostic criteria. Meanwhile, they were also divided into internal medicine treatment group (N=95) and surgery/death group (N=49) according to clinical prognosis. Four patients in the internal medicine treatment group died as they rejected the operation.

### Abdominal Ultrasound and Plain X-rays Inspection

Bedside abdominal CDUS and plain X-rays inspection were immediately performed once suspected NEC cases were found. The time interval between abdominal CDUS and X-rays inspection was less than three hours. Bedside CDUS and plain X rays inspection were performed every 6 to 12 hours according to disease conditions of infantile patients. All the abdominal ultrasound inspections used Voluson E6 Color Doppler Diasonograph (General Electric Company, Fairfield City, Connecticut, USA) with linear array transducer frequency of 7 to 12 MHz and were completed by an ultrasound specialist. X-rays inspection used SIMENSMOBILETT XP Digital DR apparatus and the results were reviewed by a radiologist.

### Statistical Analysis

All data were processed by SPSS ver. 21.0. Enumeration data with normal distribution were expressed as mean and standard deviation (SD); comparison between groups was performed by t test. Measurement data not conforming to normal distribution were expressed as median and inter-quartile range; comparison between groups was performed by rank sum test. Enumeration data were processed by chi-square test. When the minimum theoretical value was greater than 1 and less than 5, it was processed by corrected chi-square test. The results of abdominal CDUS and plain X-rays were described by risk ratio (RR) and confidence interval (CI). When P was less than 0.05 or RR 95% CI excluded 1, the difference had statistical significance.

## RESULTS

### Comparison of Basic Information between the Suspected NEC Group and Confirmed NEC Group

The differences of gestational age, birth weight, outset time, total white blood cells (TWBC), absolute neutrophil count (ANC), platelet count (PLT) and level of C Reactive Protein (CRP) between two groups had no statistical significance (Table-I).

**Table-I:** Comparison of basic information and laboratory inspection indexes between two groups.

	*Definite group (N=70)*	*Suspected group (N=74)*	*t/Z/X^2^*	*P*
Gestational age(week)	34.2±3.5	33.1±4.1	1.46	0.163
Birth weight(kg)	2.07±0.76	2.05±0.82	-0.02	0.972
Outset day(d)	11.8±7.9	14.2±8.1	-1.25	0.241
TWBC(×10^9^/L)	9.7±4.8	8.9±5.8	0.39	0.683
ANC(×10^9^/L)	5.8±4.7	6.1±4.7	-0.62	0.541
PLT(×10^9^/L)	277.2±126.2	261.2±128.1	0.61	0.573
CRP(mg/L)	5.0 (1.1,39.0)	9.5 (3.0,44.7)	-1.05	0.269

*Notes:* CRP value was expressed as [M (P25, P75)] and all other numeric values were expressed as mean±SD

### Comparison of Imaging Results of Abdominal CDUS and Plain X-rays between Suspected Group and Definite Group

The difference of dilatation of intestine, reduced inflation of intestine and bowel wall thickening in suspected NEC group had no statistical significance. In addition, abdominal ultrasounds detected out five cases of PVG while no founding by abdominal plain X-rays. According to the chi-square test of the results of abdominal ultrasound and plain X-rays in definite NEC group, the difference of PI, free intraperitoneal gas, reduced inflation of intestine and bowel wall thickening had no statistical significance. But the rate of dilatation of intestine by abdominal ultrasound was higher than plain X-rays. The difference had statistical significance. Details are shown in Table-II and III.

**Table-II:** Comparison of characteristics between abdominal plain X-rays and abdominal ultrasound in suspected NEC group [N(%)]

*Item*	*Abdominal plain X-rays (N=81)*	*Abdominal ultrasound (N=96)*	*X^2^*	*P*
Pneumatosis intestinalis	0	0		
PVG	0	5(5.2)		
Free intraperitoneal gas	0	0		
Dilatation of intestine	25(30.9)	16(16.7)	2.531	0.086
Spasticity of intestinal loop	2(2.5)	/		
Reduced inflation of intestine	25(30.9)	24(25.0)	0.634	0.462
Bowel wall thickening	23(28.4)	16(16.7)	1.892	0.148
Slowed intestinal peristalsis	/	40(41.7)		
Disappearance of intestinal peristalsis	/	6(6.3)		
Ascites	/	0		

*Notes:* / indicates no observation for the item or there is no statistical significance in difference between two groups; n indicates the number of times of abdominal plain X-rays or ultrasound inspections.

**Table-III:** Comparison of characteristics between abdominal plain X-rays and abdominal ultrasound in definite NEC group [N(%)]

*Item*	*Abdominal plain X-rays (N=91)*	*Abdominal ultrasound (N=84)*	*X^2^*	*P*
Pneumatosis intestinalis	14(15.4)	18(21.4)	0.542	0.469
PVG	15(16.5)	29(34.5)	4.361	0.036
Free intraperitoneal gas	11(12.1)	13(15.5)	0.227	0.637
Dilatation of intestine	34(37.4)	49(58.3)	4.796	0.032
Spasticity of intestinal loop	15(16.5)	/		
Reduced inflation of intestine	23(33.0)	34(40.5)	0.495	0.472
Bowel wall thickening	35(38.5)	24(28.6)	1.032	0.318
Slowed intestinal peristalsis	/	37(44.0)		
Disappearance of intestinal peristalsis	/	14(16.7)		
Ascites	/	56(66.7)		

*Notes:* / indicates no observation for the item or there is no statistical significance in difference between two groups; N indicates the number of times of abdominal plain X-rays or ultrasound inspections.

### Comparison of Imaging Results of Abdominal CDUS and Plain X-rays between Internal Medicine Treatment Group and Surgery/Death Group

This study described the relationship between the results of abdominal ultrasound and the clinical prognosis of infantile patients using RR and 95% CI. The difference of dilatation of intestine, bowel wall thickening and ascites between two groups had statistical significance. The RR of above mentioned three clinical manifestations of the surgery/death group was higher than that of the internal medicine treatment group, indicating the above three clinical manifestations could be considered as the factors for foreseeing unfavorable clinical prognosis. The RR of reduced inflation of intestine and slow down of intestinal peristalsis was less than 1, thus they were not the factors for foresting unfavorable clinical prognosis. Details are shown in Table-IV. The RR of free intraperitoneal gas and dilatation of intestine of the surgery/death group was obviously higher than that of the internal medicine treatment group; and the difference had statistical significance (P<0.05) (Table-V).

**Table-IV:** Comparison of characteristics of abdominal ultrasound between internal medicine treatment group and surgery/death group [N(%)]

*Item*	* (N=103)	** (N=55)	*X^2^ or (corrected X^2^)*	*P*	*RR*	*95%CI*
Free intraperitoneal gas	0	13(23.6)				
Pneumatosis intestinalis	10(9.7)	8(14.5)	(0.058)	0.815	1.5	0.3~4.6
PVG	20(19.4)	15(27.3)	0.846	0.357	1.4	0.8~3.1
Dilatation of intestine	31(30.1)	34(61.8)	8.407	0.000	2.1	1.2~3.2
Reduced inflation of intestine	37(35.9)	17(30.9)	0.259	0.623	0.9	0.4~1.7
Bowel wall thickening	19(18.4)	23(41.8)	6.056	0.007	2.4	1.3~4.6
Slowed intestinal peristalsis	50(48.5)	23(41.8)	0.492	0.487	0.8	0.5~1.5
Disappearance of intestinal peristalsis	10(9.7)	9(16.4)	0.483	0.476	1.8	0.7~5.4
Ascites	16(15.5)	42(76.4)	21.806	0.000	19.6	3.7~25.4

*Notes:* N indicates the number of times of abdominal plain X-rays or ultrasound inspections;

* Internal medicine treatment group;

** Surgery/death group.

**Table-V:** Comparison of characteristics of abdominal plain X-rays between internal medicine treatment group and surgery/death group [N(%)]

*Item*	*(N=110)	**(N=62)	*X^2^ or (corrected X^2^)*	*P*	*RR*	*95%CI*
Free intraperitoneal gas	0	11(17.7)				
Pneumatosis intestinalis	7(6.4)	7(11.3)	(0.452)	0.516	2	0.4~7.6
PVG	7(6.4)	9(14.5)	(1.287)	0.271	2.5	0.8~8.6
Dilatation of intestine	30(27.3)	30(48.4)	4.611	0.027	1.8	1.2~3.0
Reduced inflation of intestine	40(36.4)	15(24.2)	1.358	0.242	0.7	0.2~1.2
Bowel wall thickening	43(39.1)	32(51.6)	1.463	0.228	1.3	0.9~2.2
Spasticity of intestinal loop	9(8.2)	9(14.5)	0.717	0.403	2	0.7~6.4

*Notes:* N indicates the number of times of abdominal plain X-rays or ultrasound inspections;

* Internal medicine treatment group;

** Surgery/death group.

## DISCUSSION

NEC with high clinical incidence is a disease frequently happened to newborns. But because of its non-typical early clinical manifestations, wrong timing of surgery and concealed onset of partial cases, it severely threatens lives and health of newborns and leads to delayed treatment and high mortality.^12^ Abdominal plain X-rays were the main method for diagnosis of NEC in the past; however, X ray shows obvious defects due to its low specificity.^13^,^14^ In recent years, the diagnostic value of abdominal ultrasound for NEC diagnosis is gradually taken seriously. The application of ultrasound for early clinical diagnosis of NECs is becoming more popular with the great improvement of resolution of ultrasonic probe. It can precisely diagnose NEC symptoms of infantile patients by presenting clear images of tissue structures such as portal vein, intestinal wall and enterocoelia in ultrasonogram.^15^ Therefore abdominal ultrasound has its unique diagnostic value for NEC compared with abdominal plain X-rays.

This study indicated that abdominal CDUS was more effective in detecting PVG compared to plain X-rays during both suspected period and confirmed period, suggesting high sensitivity of abdominal CDUS. It has been generally acknowledged that there are two ways for free gas going into portal vein. As to the first way, with increase of internal pressure and dilatation of intestine, free gas in the enteric cavity infiltrates into venules on the intestinal wall because of the swelling and necrosis of intestinal mucosa and the damage of mucosal barrier, then the free gas goes back to portal vein through mesenteric vessel; the second way is direct infection of aerogen in intestine and enterocoelia.^16^ But PVG is also clinically generally seen in patients with symptoms of non-necrotizing gastrointestinal mucosa lesions, ileus, dilatation of intestine, pelvic and peritoneal abscesses, etc.^17^ Therefore, the existence of PVG needs to be determined by referring to high risk factors and clinical manifestations of patients after examination using ultrasound. In addition, during initial period of NEC, the small and tiny changes of medical conditions in intestine such as reducing of intestinal wall blood perfusion, edema, slowed intestinal peristalsis, small-scale peritoneal effusion, etc. cannot be easily found by plain X-rays; however, ultrasound inspection could obviously detect all above mentioned changes.^18^ In this study, 41.7% patients of the suspected NEC group were found slowed intestinal peristalsis. In the confirmed NEC group, 44.0% patients were found slowed intestinal peristalsis and 66.7% patients were found with ascites. Therefore, abdominal ultrasound has unique advantages in these aspects compared to abdominal plain X-rays.

Due to high fatality rate of NEC, the living quality of survived infantile patients was also a focus. In this study, abdominal ultrasound inspection revealed that the occurrence rate of dilatation of intestine, bowel wall thickening and ascites of surgery/death group was higher than that of internal medicine treatment group, suggesting those three ultrasound characteristics could be considered as the reference indexes for predicting disease severity and early unfavorable prognosis. Due to intestinal infection and functional disability of intestinal peristalsis, a great quantity of gas gathered in the enteric cavity and the intestinal wall became thinner; and in such a condition, enterobrosis was more likely to be induced. Abdominal plain film suggested that, the detection rate of dilatation of intestine was higher in the surgery/death group. Thus dilatation of intestine could be considered as the risk factor for surgery or death. Thus abdominal ultrasound was more sensitive in detecting dilatation.

The results of abdominal ultrasound inspection indicated that ascites was a risk factor for surgery/death because this factor might correlated to enterobrosis. In this study, abdominal ultrasound or plain X-rays suggested that, all the infantile patients with free intraperitoneal gas needed surgical treatment or found dead, which was consistent with the result reported in the study made by Silva et al.^19^ Besides, their study indicated ascites was the risk factor for surgery/death. In a foreign research, detecting of spasticity of intestinal loop indicated unfavorable prognosis.^20^ But the difference of our centre’s research result had no statistical significance, which was considered to be correlated to the low rate of reexamination of abdominal plain X-rays in our centre. Our study also indicated the difference of occurrence rate of PVG of internal medicine treatment group and surgery/death group had no statistical significance and PVG was neither the surgical indication nor the index for foreseeing unfavorable clinical prognosis, which confirmed the result of Sharma et al study.^21^ Lack of general clinical reference standards, it was difficult to compare the sensitivity of abdominal ultrasound and plain X-rays to above characteristics. But generally speaking, the imaging results of abdominal ultrasound could be considered as the reference index of clinical prognosis for infantile patients with NEC.

## CONCLUSION

Abdominal ultrasound is important to both NEC diagnosis and prediction of clinical unfavorable prognosis. Therefore, for infantile patients with suspected or confirmed NEC, we can monitor them dynamically and adjust treatment methods with the changes in medical conditions to lower surgical and death rate and improve prognosis. But there were few re-examinations of abdominal ultrasound and plain X-rays for the cases included in our study so that we could not find the changes of free intra peritoneal gas, PVG, PI, etc. in time, which reduced the diagnosis to some extent. On the other hand, these two kinds of inspections hardly proceeded at the same time so we could not precisely acknowledge the time sequence of changes of characteristics in both inspections, which limited overall evaluation of the superiority of abdominal CDUS in evaluating NEC. In future clinical works, time tables for abdominal CDUS and plain X-rays examination and re-examination are suggested to be used.
